# Ten Simple Rules for a Good Poster Presentation

**DOI:** 10.1371/journal.pcbi.0030102

**Published:** 2007-05-25

**Authors:** Thomas C Erren, Philip E Bourne

Posters are a key component of communicating your science and an important element in a successful scientific career. Posters, while delivering the same high-quality science, offer a different medium from either oral presentations [1] or published papers [2], and should be treated accordingly. Posters should be considered a snapshot of your work intended to engage colleagues in a dialog about the work, or, if you are not present, to be a summary that will encourage the reader to want to learn more. Many a lifelong collaboration [3] has begun in front of a poster board. Here are ten simple rules for maximizing the return on the time-consuming process of preparing and presenting an effective poster.

## Rule 1: Define the Purpose

The purpose will vary depending on the status and nature of the work being presented, as well as the intent. Some posters are designed to be used again and again; for example, those making conference attendees aware of a shared resource. Others will likely be used once at a conference and then be relegated to the wall in the laboratory. Before you start preparing the poster, ask yourself the following questions: What do you want the person passing by your poster to do? Engage in a discussion about the content? Learn enough to go off and want to try something for themselves? Want to collaborate? All the above, or none of the above but something else? Style your poster accordingly.

## Rule 2: Sell Your Work in Ten Seconds

Some conferences will present hundreds of posters; you will need to fight for attention. The first impressions of your poster, and to a lesser extent what you might say when standing in front of it, are crucial. It is analogous to being in an elevator and having a few seconds to peak someone's interest before they get off. The sad truth is that you have to sell your work. One approach is to pose your work as addressing a decisive question, which you then address as best you can. Once you have posed the question, which may well also be the motivation for the study, the focus of your poster should be on addressing that question in a clear and concise way.

## Rule 3: The Title Is Important

The title is a good way to sell your work. It may be the only thing the conference attendee sees before they reach your poster. The title should make them want to come and visit. The title might pose a decisive question, define the scope of the study, or hint at a new finding. Above all, the title should be short and comprehensible to a broad audience. The title is your equivalent of a newspaper headline—short, sharp, and compelling.

## Rule 4: Poster Acceptance Means Nothing

Do not take the acceptance of a poster as an endorsement of your work. Conferences need attendees to be financially viable. Many attendees who are there on grants cannot justify attending a conference unless they present. There are a small number of speaking slots compared with attendees. How to solve the dilemma? Enter posters; this way everyone can present. In other words, your poster has not been endorsed, just accepted. To get endorsement from your peers, do good science and present it well on the poster.

## Rule 5: Many of the Rules for Writing a Good Paper Apply to Posters, Too

Identify your audience and provide the appropriate scope and depth of content. If the conference includes nonspecialists, cater to them. Just as the abstract of a paper needs to be a succinct summary of the motivation, hypothesis to be tested, major results, and conclusions, so does your poster.

## Rule 6: Good Posters Have Unique Features Not Pertinent to Papers

The amount of material presented in a paper far outweighs what is presented on a poster. A poster requires you to distill the work, yet not lose the message or the logical flow. Posters need to be viewed from a distance, but can take advantage of your presence. Posters can be used as a distribution medium for copies of associated papers, supplementary information, and other handouts. Posters allow you to be more speculative. Often only the titles or at most the abstracts of posters can be considered published; that is, widely distributed. Mostly, they may never be seen again. There is the opportunity to say more than you would in the traditional literature, which for all intents and purposes will be part of the immutable record. Take advantage of these unique features.

## Rule 7: Layout and Format Are Critical

Pop musician Keith Richards put the matter well in an interview with *Der Spiegel* [4]: “If you are a painter, then the most important thing is the bare canvas. A good painter will never cover all the space but will always leave some blank. My canvas is silence.” Your canvas as poster presenter is also white space. Guide the passerby's eyes from one succinct frame to another in a logical fashion from beginning to end. Unlike the literature, which is linear by virtue of one page following another, the reader of a poster is free to wander over the pages as if they are tacked to the poster board in a random order. Guide the reader with arrows, numbering, or whatever else makes sense in getting them to move from one logical step to another. Try to do this guiding in an unusual and eye-catching way. Look for appropriate layouts in the posters of others and adopt some of their approaches. Finally, never use less than a size 24 point font, and make sure the main points can be read at eye level.

## Rule 8: Content Is Important, but Keep It Concise

Everything on the poster should help convey the message. The text must conform to the norms of sound scientific reporting: clarity, precision of expression, and economy of words. The latter is particularly important for posters because of their inherent space limitations. Use of first-rate pictorial material to illustrate a poster can sometimes transform what would otherwise be a bewildering mass of complex data into a coherent and convincing story. One carefully produced chart or graph often says more than hundreds of words. Use graphics for “clear portrayal of complexity” [5], not to impress (and possibly bewilder) viewers with complex artistry. Allow a figure to be viewed in both a superficial and a detailed way. For example, a large table might have bold swaths of color indicating relative contributions from different categories, and the smaller text in the table would provide gritty details for those who want them. Likewise, a graph could provide a bold trend line (with its interpretation clearly and concisely stated), and also have many detailed points with error bars. Have a clear and obvious set of conclusions—after the abstract, this is where the passerby's eyes will wander. Only then will they go to the results, followed by the methods.

## Rule 9: Posters Should Have Your Personality

A poster is a different medium from a paper, which is conventionally dry and impersonal. Think of your poster as an extension of your personality. Use it to draw the passerby to take a closer look or to want to talk to you. Scientific collaboration often starts for reasons other than the shared scientific interest, such as a personal interest. A photo of you on the poster not only helps someone find you at the conference when you are not at the poster, it can also be used to illustrate a hobby or an interest that can open a conversation.

## Rule 10: The Impact of a Poster Happens Both During and After the Poster Session

When the considerable effort of making a poster is done, do not blow it on presentation day by failing to have the poster achieve maximum impact. This requires the right presenter–audience interaction. Work to get a crowd by being engaging; one engaged viewer will attract others. Don't badger people, let them read. Be ready with Rule 2. Work all the audience at once, do not leave visitors waiting for your attention. Make eye contact with every visitor.

Make it easy for a conference attendee to contact you afterward. Have copies of relevant papers on hand as well as copies of the poster on standard-sized paper. For work that is more mature, have the poster online and make the URL available as a handout. Have your e-mail and other demographics clearly displayed. Follow up with people who come to the poster by having a signup sheet.

The visitor is more likely to remember you than the content of your poster. Make yourself easy to remember. As the host of the work presented on the poster, be attentive, open, and curious, and self-confident but never arrogant and aggressive. Leave the visitors space and time—they can “travel” through your poster at their own discretion and pace. If a visitor asks a question, talk simply and openly about the work. This is likely your opportunity to get feedback on the work before it goes to publication. Better to be tripped up in front of your poster than by a reviewer of the manuscript.

Good posters and their presentations can improve your reputation, both within and outside your working group and institution, and may also contribute to a certain scientific freedom. Poster prizes count when peers look at your resume.

These ten rules will hopefully help you in preparing better posters. For a more humorous view on what not to do in preparing a poster, see [6], and for further information, including the opportunity to practice your German, see [7]. 
